# Household food insecurity and mental distress among pregnant women in Southwestern Ethiopia: a cross sectional study design

**DOI:** 10.1186/s12884-015-0699-5

**Published:** 2015-10-08

**Authors:** Mulusew G. Jebena, Mohammed Taha, Motohiro Nakajima, Andrine Lemieux, Fikre Lemessa, Richard Hoffman, Markos Tesfaye, Tefera Belachew, Netsanet Workineh, Esayas Kebede, Teklu Gemechu, Yinebeb Tariku, Hailemariam Segni, Patrick Kolsteren, Mustafa al’Absi

**Affiliations:** 1 Population and Family Health, Jimma University, Jimma, Ethiopia; 2 Department of Epidemiology, Jimma University, Jimma, Ethiopia; 3 Duluth Medical Research Institute, Department of Bio behavioral Health and Population Sciences, University of Minnesota Medical School, Duluth, MN USA; 4 Department of Horticulture and Plant Sciences, Jimma University, Jimma, Ethiopia; 5 Department of Psychiatry, Jimma University, Jimma, Ethiopia; 6 Department of Internal Medicine, Jimma University, Jimma, Ethiopia; 7 Department of Pediatrics and Child Health, Jimma University, Jimma, Ethiopia; 8 Department of Psychology, Jimma University, Jimma, Ethiopia; 9 Department of Chemistry, Jimma University, Jimma, Ethiopia; 10 Department of Obstetrics and Gynecology, Jimma University, Jimma, Ethiopia; 11 Department of Food Safety and Food Quality, Ghent University, CoupureLinks, Ghent, Belgium

**Keywords:** Food insecurity, Mental distress, Pregnant women, Ethiopia

## Abstract

**Background:**

There are compelling theoretical and empirical reasons that link household food insecurity to mental distress in the setting where both problems are common. However, little is known about their association during pregnancy in Ethiopia.

**Methods:**

A cross-sectional study was conducted to examine the association of household food insecurity with mental distress during pregnancy. Six hundred and forty-two pregnant women were recruited from 11 health centers and one hospital. Probability proportional to size (PPS) and consecutive sampling techniques were employed to recruit study subjects until the desired sample size was obtained. The Self Reporting Questionnaire (SRQ-20) was used to measure mental distress and a 9-item Household Food Insecurity Access Scale was used to measure food security status. Descriptive and inferential statistics were computed accordingly. Multivariate logistic regression was used to estimate the effect of food insecurity on mental distress.

**Results:**

Fifty eight of the respondents (9 %) were moderately food insecure and 144 of the respondents (22.4 %) had mental distress. Food insecurity was also associated with mental distress. Pregnant women living in food insecure households were 4 times more likely to have mental distress than their counterparts (COR = 3.77, 95 % CI: 2.17, 6.55). After controlling for confounders, a multivariate logistic regression model supported a link between food insecurity and mental distress (AOR = 4.15, 95 % CI: 1.67, 10.32).

**Conclusion:**

The study found a significant association between food insecurity and mental distress. However, the mechanism by which food insecurity is associated with mental distress is not clear. Further investigation is therefore needed to understand either how food insecurity during pregnancy leads to mental distress or weather mental distress is a contributing factor in the development of food insecurity.

## Background

Mental distress is becoming one of the major contributors to the global burden of diseases in developing countries [1, 2]. Mental distress is defined as a collection of mental problems that may not fall into standard diagnostic criteria and are characterized by symptoms of sleeplessness, exhaustion, irritability, poor memory, difficulty in concentrating, and somatic complaints [3].

Numerous epidemiologic studies have revealed that maternal distress during pregnancy is a public health concern in Sub Saharan African [4–8]. It has been associated with low infant birth weight, impaired postnatal growth, increased frequency of infant diarrhea [9, 10], under nutrition, stunted growth, and poorer cognitive development [11, 12]. Previous study done in Ethiopia also showed that maternal distress was associated with child growth and low birth weight [10, 13].

If public health measures aim to improve the mental health status of pregnant women, there must be empirical data revealing the social, behavioral, economic and cultural factors associated with mental distress [14–17]. Among these, food insecurity is one of the determinants that are known to increase risk for mental distress [5, 18–21]. Food insecurity is defined as either the lack of nutritionally adequate and safe food or a limited ability to acquire acceptable food in socially acceptable ways [22]. It is a process that may start with household members being worried about not being able to access food followed by sacrificing the quality of their diet and then eventually reducing the amount of calories consumed. This usually occurs when regular access to adequate and nutritious food is limited [23, 24].

Food insecurity has been identified as a public health issue for women living in low-income households which results in poorer health outcomes [25]. For example, food insecurity is associated with decreased self-rated health status among adults and children [18, 26–28], depression and anxiety in mothers [29] reduced micronutrient intake, decreased fruit and vegetable consumption[30, 31], overweight [32], poor child physical growth [25, 33] low birth weight & elevated prenatal depressive symptoms [34]. The association between maternal depression and food insecurity has been also reported [5, 29–32]. But, few studies have examined the association between mental distress and food insecurity among pregnant women [35].

In Ethiopia both mental illness and food insecurity are reported. While previous research has shown an association between mental health and food insecurity among adults, non-pregnant women and children [7, 10, 13, 28, 36, 37], there is scant literature studying their relationship during pregnancy in Ethiopia. This study tried to answer the following two questions: One, what is the magnitude of both mental distress and food insecurity among pregnant women? Two is there an association between food insecurity and mental distress among pregnant women? Thus, the purpose of this study was to document the magnitude of food insecurity and mental distress and to examine their association during pregnancy. These findings can be used for program planners and researcher to further study the link between the two in southwestern Ethiopia.

## Methods

### Study setting

A facility-based cross-sectional study was conducted in Jimma Zone, one of the 20 administrative zones in Oromia Regional State, southwest Ethiopia. According to the Central Statistical Authority [38] 2.7 million people live in Jimma Zone on an area of 15,569 km^2^ with a population density of 159.69 persons per km^2^. Of these, 1.23 million are women. An estimated 31,050 women become pregnant every year and antenatal care coverage in the zone is 64.3 percent. There are three hospitals and 84 primary health centers in Jimma zone where pregnant mothers can receive antenatal care services. There are 12 public health facilities affiliated with Jimma university within the radius of 70 km for community based education program, research and services. Between the months of June and August 2013, a total of 2,987 pregnant women were on antenatal care (ANC) follow up at the 12 health facilities selected for this study [39].

### Sample size and sampling

A single population proportion formula was used to estimate 660 pregnant women to be included in the sample. Assumptions for calculating the sample size were the degree of confidence interval (95 %; Z_1-α/2_ = 1.96), the estimated magnitude of mental distress among pregnant women (P = 50 %), a 4 % degree of precision, and a non-response rate of 10 %. A total of 11 health centers and one hospital were selected to sample the study subjects. These facilities were chosen purposefully based on their previous affiliation with the Jimma University Community Based Education Training Program (CBTP). Probability proportional to size sampling (PPS) techniques was employed to assign the number of pregnant women to be interviewed from each selected health center and hospital. All pregnant women coming for ANC services during the data collection period were taken as the source population for the study. Finally, a consecutive sampling technique was used to identify the study subjects from each of the health facilities until the desired sample size was obtained. Seriously ill pregnant women were excluded from the study and referred to the respective hospital.

### Tools and measurements

The instruments used for data collection were adapted from earlier studies and WHO guidelines [30, 40–48] and translated from English into the two most commonly spoken languages in the study setting (Afan Oromo and Amharic) by two fluent linguists (from the university Language and Literature Department). Each translation was then translated back by another person (linguists from the English Department) to ensure its consistency. Before the actual survey, the final translated questionnaires were pre tested on 5 % of the sample at two different health institutions (one urban and the other rural) so that coherence, wording, sequencing and consistencies of all questions were amended accordingly. The result of this pretest was not included in the main analyses. Two days of intensive training on how to approaches the clients, interview techniques, ethical consideration and how to refer any mothers that needed help. Finally, exit interviews were conducted by trained data collectors at each of the 12 health facilities immediately after the mothers had received their ANC services. Field supervisors and the research team supervised the data collection process. Supervisors also checked the consistency of data before submission to the data manager. Ethical clearance was obtained from the Jimma University (JU) ethical review board. The participants were asked for their oral consent after the purpose of the study was clearly communicated. Confidentiality was ensured for each study participants.

### Questionnaires to measure mental distress

Mental distress was measured using The Self Reporting Questionnaire (SRQ-20). The SRQ-20 is a screening instrument developed by the World Health Organization (WHO) to assess the level of symptoms of overall mental distress one month preceding the survey [42]. Scores range from 0 to 20, with higher scores representing more severe mental distress. The SRQ has been used in several previous studies exploring the relationship between maternal psychological wellbeing and infant health [42, 46]. In developing countries, cutoff scores of ≥6, 7, 8, 4 and 10 have been used for identifying cases of mental distress [40, 42, 46, 48]. However, in this study we report the proportion of women scoring SRQ ≥7 or greater to indicate mental distress. The SRQ-20 has been validated in Ethiopia for measuring mental distress among rural pregnant women and the recommended cutoff points greater or equal to 7 have specificity of 62 % and sensitivity of 68.4 % [40]. If the scoring was <7, we coded “0” for no mental distress and if it was greater or equals to 7, we coded “1” to indicate presence of mental distress.

### Questionnaire to measure household food insecurity access scale

Household food insecurity access was measured using items from the validated Household Food Insecurity Access Scale (HFIAS) that was specifically developed for use in developing countries [41, 43–45]. The HFIAS consists of 9 items specific to an experience of food insecurity occurring within the previous four weeks. Each respondent indicated whether they had encountered the following due to lack of food or money to buy food in the last one month: (1) worried about running out of food, (2) lack of preferred food, (3) the respondent or another adult had limited access to a variety of foods due to a lack of resources (4) forced to eat un preferred food due to lack of resources, (5) eating smaller portions, (6) skipping meals, (7) the household ran out of food, (8) going to sleep hungry, and (9) going 24 hours without food. Endorsed items are then clarified with reported estimates of the frequency of food insecurity (rarely, sometimes, and often). Scores range from 0 to 27 where higher scores reflect more severe food insecurity and lower scores represent less food insecurity. To determine the status of food insecurity the average HFIAS score (dividing the sum of Household score by number of household in the sample) was computed and then household food insecurity access prevalence (HFIAP) categories (food secure, mild, moderately and severely food insecure) was generated. But, since none of the mothers reported mild and severely food insecure households, HFIAP was only categorized in to two conditions [44].

### Data management and analysis

The data were entered, cleaned, and analyzed using STATA version 12 for Microsoft Windows. Descriptive statistics, bivariate and multivariate logistic regression analyses were computed to examine the relationship between the explanatory variables and mental distress. Assuming a linear relationship between independent and dependent variables, the binary form of the dependent variable was coded as “1” for mental distress and “0” for not distressed. First binary logistic regression analyses were conducted between each and separate explanatory variables with the outcome of our interest (mental distress) (Model I) and reported using crude Odds Ratios. Finally, all significant variables(P < 0.05) during the bivariate analyses were chosen for multivariate logistic regression modeling using forward selection method to explore the association of food insecurity with mental distress by controlling for other confounding variables such as age, occupation, monthly income, and ownership of agricultural land (Model II). Adjusted odds ratios (AOR) and their 95 % confidence intervals (CI) were presented as indicators of strength of association. A p-value of 0.05 or less was used to determine the cut-off points for statistical significance.

## Results

### Socio demographic characteristics

A total of 642 pregnant women agreed to participate in the study resulting in a response rate of 97.3 %. The mean (± SD) age of the mother was 25.5(± 4.9). The majority of the women (67.1 %) were between the ages of 20 and 29 years old. A total of 91 % of them were married. Just over half of them were from urban areas (58.7 %) and most (69.6 %) of them were Muslim by religion. About one quarter of the women were illiterate (26.8 %). Half of the women had completed primary school or were able to read and write (26.6 % and 23.5 % respectively). The mean (±SD) household size was 3.9 (±1.6) (Table 1).

**Table 1:** Socio demographic characteristics of respondents in Jimma Zone, southwest Ethiopia, 2013

Variables	Categories	Number	Percentage
Residence	Urban	377	58.7
	Rural	265	41.3
Household size	≤4	435	67.8
	>4	207	32.2
Age of respondents	15-24	277	43.2
	25-34	318	49.5
	≥35	47	7.3
Occupation	Farmer	172	26.8
	Gov. employee	107	16.7
	Merchant	83	12.9
	Daily laborer	66	10.3
	Others^a^	214	33.3
Marital status	Married	584	91.0
	Others^b^	58	9.0
Ethnicity	Oromo	494	76.9
	Dawuro	32	5.0
	Amhara	75	11.7
	Others^c^	41	6.4
Religion	Muslim	447	69.6
	Orthodox	141	22.0
	Others^d^	54	8.4
Educational status	Illiterate	172	26.8
	Read and write	151	23.5
	Primary school	171	26.6
	Secondary school	60	9.3
	College& above	88	13.7
Number of children <5	0	234	36.4
	1	395	61.5
Food insecurity status	2	13	2.0
	Food secure	584	91.0
	Food insecure	58	9.0

^a^Students, housewife, maid workers, ^b^single, widowed, divorced. ^C^ Wolaita, Kullo,Tigray, Yam^,^^d^Catholics, protestant

### Socioeconomic and household wealth

Just over one quarter of the women reported a household monthly income less than 1,000 Ethiopian Birr (ETB). One hundred seventy-two (26.8 %) of women were involved in farming, and 274 (42.7 %) of women owned agricultural land. Three hundred forty-six (53.9 %) of the respondents did not have electricity and 65 (10.1 %) of the respondents did not have the use of a latrine. Most respondents (89.1 %) used firewood or other agricultural products as fuel for cooking. Water was provided by access to pipe water or tap water in 61.1 % of the households while 39 % depend on spring, river or well water. While most had a radio (72 %) and a cell phone (77.3 %) in the home, other household items such as television, land line phones and refrigerators were uncommon (30.2 %, 6.2 % and 11.8 % respectively) (Table 2).

**Table 2:** Socioeconomic and household wealth of respondents, southwest Ethiopia, 2013

Household effects	Category	Number	Percentage
Electricity	Yes	296	46.1
	No	346	53.9
Television	Yes	194	30.2
	No	448	69.8
Radio	Yes	462	72.0
	No	180	28.0
Telephone- fixed	Yes	40	6.2
	No	602	93.8
Mobile telephone	Yes	496	77.3
	No	146	22.7
Refrigerator	Yes	76	11.8
	No	566	88.2
Own agricultural Land	Yes	274	42.7
	No	368	57.3
Latrine	Yes	577	89.9
	No	65	10.1
Type of fuel	Electricity	18	2.8
	Wood&Agri crops.	572	89.1
	Charcoal	48	7.5
Source of water supply	Others(e.g.kerosene)	4	0.8
	Pipe water	195	30.4
	Tap water	197	30.7
	spring	202	31.5
	Dig well	23	3.6
Family-monthly income^e^	Protected river water	25	3.9
	≤500	70	10.9
	500-999	106	16.5
Transportation	≥1000	466	72.6
Bicycle	Yes	40	6.2
	No	602	93.8
Animal drawn cart	Yes	34	5.3
Bajaj^f^	No	608	94.7
	Yes	13	2.0
	No	629	98.0
Car	Yes	7	1.1
	No	635	98.9

^*e*^National currency in Birr, 3/27/2014 exchange rate 1 US Dollar = 19.37 Birr, 1 Euro = 27.627 Birr ^f^Bajaj is a three wheel drive motor bike owned by household used for transportation

### Prevalence of household food insecurity and mental distress

Internal consistency of the Household Food Insecurity Access Scale was strong (Cronbach’s alpha = 0.96). The reliability coefficient (Cronbach’s Alpha) for the SRQ-20 was 0.858, showing very good internal consistency of the items. Fifty-eight (9 %) of the respondents were living in food insecure households and twenty-two percent of the pregnant women had mental distress (22.4 %). The prevalence of mental distress was higher among pregnant women living in a food insecure environment (48.3 %) when compared to those living in food secured households (19.9 %).

### Association between food insecurity and mental distress

Table 3 reports the association between food insecurity and mental distress. Pregnant women living in urban areas were more likely to have mental distress when compared to their rural counter parts (COR = 3.01, 95 % CI =1.19, 7.60). Lower monthly income (≤500 ETB) was associated with mental distress (COR = 1.94, 95 % CI = 1.12, 3.35). Pregnant women living in food insecure households were more likely to have mental distress (COR = 3.77, 95 % CI = 2.17, 6.55). Multivariate logistic regression modeling analysis using the forward selection method was used to investigate the independent association of food insecurity with mental distress. The result showed that pregnant women living in a food insecure household were 4.15 times more likely to have mental distress (AOR = 4.15, 95 % CI = 1.67, 10.32). In addition, this study reveals mental distress is also associated with a family history of mental illness (AOR = 5.85, 95 % CI = 2.23, 15.34) and intimate partner violence (AOR = 3.20, 95 % CI = 1.43, 7.13)

**Table 3:** Factors associated with mental distress among pregnant women in Jimma Zone, southwest Ethiopia, 2013

Variables	Categories	*COR(95 % CI)*	*AOR(95 % CI)*
Residence	Urban	1. 74(1.17,2.58)	3.01(1.19,7.60)
	Rural	1	1
HH size	>4	0.80 (0.53,1.20)	2.26(1.25,4.19)
	≤4	1	1
Age	≥35	1.82(0.69,4.77)	3.44(0.91,12.96)
	30-34	1.44(0.62,3.35)	2.22(0.71,6.91)
	25-29	1.49(0.67,3.27)	1.67(0.58,4.81)
	20-24	1.26(0.57,2.77)	1.53(0.55,4.30)
	15-19	1	1
Occupation	Gove. employee	0.81(0.40,1.62)	0.33(0.09,1.26)
	Merchant	1.82(0.95,3.46)	1.04(0.31,3.50)
	Daily laborer	2.51(1.29,4.85)	1.03(0.26,3.94)
	Others	2.14(1.29,3.55)	1.65(0.62,4.41)
	Farmer	1	1
Monthly income	≤500	1.94(1.12,3.35)	0.76(0.32,1.82)
	501-999	1.35(0.83,2.21)	0.96(0.44,2.07)
	≥1000	1	1
Marital status	In Marriage	0.37(0.21,0.64)	0.05(0.01,0.33)
	Not in marriage	1	1
Land ownership	Yes	0.71(0.47,1.08)	1.23(0.68,2.22)
	No	1	1
Previous Hx of IPV	Yes	3.92(2.38,6.46)	3.20(1.43,7.13)
	No	1	1
Current Hx of IPV	Yes	1.95(1.10,3.48)	0.48(0.17,1.31)
	No	1	1
Food insecurity	Food insecure	3.77(2.17,6.55)	4.15(1.67,10.32)
	Food secure	1	1
FamHx of mental disorder	Yes	3.90(2.10,7.28)	5.85(2.23,15.34)
	No	1	1
Currently chewed khat	Yes	1.35(0.86,2.12)	2.25(1.05,4.83)
	No	1	1

COR- Crude Odds Ratios, AOR- Adjusted Odds Ratio, IPV-Intimate Partner Violence

## Discussion

This is a multi-center, facility-based cross sectional study conducted to determine the magnitude of food insecurity and its correlates with mental distress during pregnancy among women following antenatal care. Previously, there was limited information related to common mental distress among pregnant women in this study setting. Moreover, findings from previous studies on the general adult community, adolescents and children [14, 18, 27, 29, 33]. To the best of our knowledge, there has been limited study of antenatal mental distress in Ethiopia; one study that measured the prevalence of postnatal maternal and paternal symptoms among adult women revealed rates of 37 % for anxiety and 19.9 % for depression respectively [7, 10, 11]. Therefore, the present study addresses the information gap regarding the prevalence of food insecurity and its correlate with mental distress among pregnant women and therefore expands the scientific literature in this area.

In this study the current prevalence of food insecurity was 9 % and mental distress was 22.4 %. The magnitude of household food insecurity in this study was very small when compared with a similar previous study [36, 37]. Dibaba et al., 2013 noted that 41 % of women in that study reported food insecurity [7]. There are three possible reasons that might explain this observed difference. The first reason is the difference in the versions of the HFIAS used as a measure of food insecurity. We used 9 items and other studies have used a 6-item measurement scale. The second reason is the difference in study design. The other studies used a cohort and community-based cross sectional sampling as well as a different study population (i.e. the other studies used pregnant women in a community based design and a female adolescent population). This study focused on pregnant women in a facility-based design. The other difference might be due to the seasonal effect on household food insecurity status, i.e. there are times when most households run out of food and food insecurity tends to be high during those seasons.

The magnitude of mental distress was 22.4 % in the present study, which is approximately consistent with a previous study conducted in Ethiopian communities in which about 22.7 % of those subjects reported mental distress [15, 49, 50]. A similar study in southwest Ethiopia reported the magnitude of antenatal depression as 19.9 %, which is within the range of findings reported from Sub-Saharan Africa and other developing countries [15, 16, 32, 43]. However, the rate of mental distress in the present study is higher than the 11.7 % of adults prevalence reported from Addis Ababa [14], the 12 % from southern Ethiopia [49], and the 17 % rate reported from Butajira, south-central Ethiopia [13] and it was also very high when compared to the reported 12 % prevalence of prenatal depression in developed countries [51]. The difference in prevalence may be due to the tools used to measure the psychological morbidity or the cut-off points used. Some of these studies used higher/lower cut-off points to measure SRQ. The difference in prevalence might also be due to a difference in the study populations. Pregnant women are challenged by different physiologic and psychological stressors [7, 52–55], and the differential presence of hormones during pregnancies might contribute to the reported distress [56]. However, the high prevalence of maternal depression in poor countries may be related to women’s exposure to several depression-related risk factors [52–54]. For instance, in settings of socio-economic adversity, there is substantial evidence for a relationship between intimate partner violence, lack of social support as well as poverty, and maternal mental distress during their pregnancies [57–60]. This study also reports that pregnant women who experienced intimate partner violence have significantly associated maternal distress. This is consistent with studies from other developing regions where intimate partner violence during pregnancy was reported to be significantly associated with poor mental health symptoms [54, 55]. The present study also revealed that urban pregnant women were more likely to report mental distress compared to their rural counterparts. The pressure associated with urban living where there is high economic vulnerability and poor mental health status might explain this. For example, a study conducted in rural Malawi utilizing the SRQ-20 showed that social circumstances and type of employment among women influenced psychological conditions and their expression [61]. The authors recommend that further studies be conducted in order to explore the relationship between urbanization and mental distress.

The present study showed a significant association between food insecurity and mental distress. The magnitude of food mental distress was higher (48.3 %) among pregnant women living in a food insecure household compared with those from food secure households (19.9 %). Other studies have also indicated that the effects of food insecurity extend beyond the nutritional effects [10, 11, 18, 62]. This is consistent with other studies documenting a close association between food insecurity and mental distress [5–7, 18, 20, 22, 30] that showed food insecurity to be associated with depressive symptoms. The mechanism by which food insecurity leads to mental distress is difficult to determine using a cross sectional design. Nonetheless, available evidence suggests that there are biological pathways and psycho social pathways where food insecurity leads to mental distress [52]. In general, despite differences in study subject characteristics and different cut-off points used to define mental distress and food insecurity, our study findings is in general agreement with previous reports of research completed in Ethiopia [7, 36, 53]. The present study has some methodological strength. There was a high response rate; the study was also facilities-based, which helped us include study participants with varied socio economic and cultural backgrounds. This study could also give an insight for policy makers and program planners to device appropriate strategies for prevention of food insecurity and mental health. However, there are also some study limitations. First, given that this is a cross-sectional study, it is not possible to establish causal relationships. The cross-sectional nature of our data did not enable us to show the direction of the association between household food insecurity and mental distress. Therefore, prospective longitudinal studies are needed in the study setting among pregnant women to understand better the bidirectional relationship. Secondly, mental distress is based on self-report and thus is subject to non-systematic errors in recall and systematic nondisclosure. Both types of errors may have led to some misclassification in our study. Thirdly, study findings may not be generalized to the broader pregnant population since our study was limited to only women presenting to the ANC at health care facilities. In a country such as Ethiopia, where a significant proportion of pregnant women do not have antenatal follow-up, the findings of the present study cannot be generalized to the broader population of pregnant women in the community. There are also other exogenous variables responsible for mental distress but these were not assessed by this study. Other studies may be required to understand different factors responsible for the cause of mental distress among pregnant women at the individual, community and societal level. In addition, lack of qualitative data to further explore their relationship is the major limitation to this study. More importantly, while this study reflects the public health importance of food security and mental distress, to quantify the prevalence of mental distress we used a cut off ≥7; there are different cut offs used by other researchers. We chose our particular cut-off because previous validation studies in Ethiopia using this cut point yielded an optimum SRQ cut-off score to determine the case for mental distress with 68.4 % sensitivity and 62 % specificity. In addition, studying at health facilities may have skewed the responses to affirmative responses for attempts to elicit help, rather than a manifestation of emotional distress [40].

## Conclusion

This study found a 9 % prevalence of food insecurity and a 22.4 % prevalence of mental distress among pregnant women. The prevalence of mental distress is reported to be 48.3 % and 19.9 % among food insecure and food secure households respectively. Moreover, the results showed that there was an association between food insecurity and mental distress. However, the mechanism by which food insecurity is associated with mental health was not clearly known. Thus, further study is recommended to determine if food insecurity during pregnancy produces mental distress or weather mental distress is a contributing factor in the development of food insecurity. Future research should also investigate the impact of food insecurity and mental distress on pregnancy outcomes, fetal & child growth and development.

## Abbreviations

AOR: Adjusted odds ratio
ANC: Ante natal care
CBTP: Community based training program
CI: Confidence interval
COR: Crude odds ratio
ETB: Ethiopian Birr
HFIAS: Household food insecurity access scale
HFIAP: Household food insecurity access prevalence
JU: Jimma University
KRP: Khat research program
SD: Standard deviation
SRQ: The self-rated Questionnaire
USA: United State of America
WHO: World Health Organization
